# Supplementary material for: Heparin-based hydrogel scaffolding alters the transcriptomic profile and increases the chemoresistance of MDA-MB-231 triple-negative breast cancer cells
Source: Biomater Sci. 2020 Feb 13;8(10):2786–96. doi: 10.1039/c9bm01481k (PMC7497406; doi:10.1039/c9bm01481k)
Supplement: Supplementary file 2 [file BM-008-C9BM01481K-s002.zip › Supplementary File 4/HP-B hydrogel + EGF (s)vControl/Pathways/my_analysis.Gsea.1545201170987/HALLMARK_DNA_REPAIR.html]

Details for gene set HALLMARK\_DNA\_REPAIR[GSEA]

|  || Dataset | expr.class.cls#HYDEGF\_versus\_CONTROL.class.cls#HYDEGF\_versus\_CONTROL\_repos |
| Phenotype | class.cls#HYDEGF\_versus\_CONTROL\_repos |
| Upregulated in class | CONTROL |
| GeneSet | HALLMARK\_DNA\_REPAIR |
| Enrichment Score (ES) | -0.26052335 |
| Normalized Enrichment Score (NES) | -1.1936535 |
| Nominal p-value | 0.10576923 |
| FDR q-value | 0.16478382 |
| FWER p-Value | 0.936 |
Table: GSEA Results Summary

  

Fig 1: Enrichment plot: HALLMARK\_DNA\_REPAIR      
 Profile of the Running ES Score & Positions of GeneSet Members on the Rank Ordered List

  

| PROBE | DESCRIPTION (from dataset) | GENE SYMBOL | GENE\_TITLE | RANK IN GENE LIST | RANK METRIC SCORE | RUNNING ES | CORE ENRICHMENT || 1 | DDB2 | na |  |  | 266 | 2.430 | 0.0073 | No |
| 2 | RAD52 | na |  |  | 353 | 2.305 | 0.0230 | No |
| 3 | DGCR8 | na |  |  | 415 | 2.217 | 0.0392 | No |
| 4 | ERCC2 | na |  |  | 455 | 2.172 | 0.0562 | No |
| 5 | ERCC3 | na |  |  | 582 | 2.035 | 0.0675 | No |
| 6 | TAF6 | na |  |  | 826 | 1.804 | 0.0705 | No |
| 7 | TARBP2 | na |  |  | 996 | 1.688 | 0.0765 | No |
| 8 | ELL | na |  |  | 1293 | 1.517 | 0.0742 | No |
| 9 | POLA2 | na |  |  | 1347 | 1.495 | 0.0845 | No |
| 10 | POLD1 | na |  |  | 1350 | 1.493 | 0.0975 | No |
| 11 | POLR2A | na |  |  | 1912 | 1.289 | 0.0794 | No |
| 12 | POLL | na |  |  | 2495 | 1.130 | 0.0588 | No |
| 13 | LIG1 | na |  |  | 2535 | 1.119 | 0.0665 | No |
| 14 | SAC3D1 | na |  |  | 2564 | 1.111 | 0.0748 | No |
| 15 | TAF1C | na |  |  | 2611 | 1.101 | 0.0820 | No |
| 16 | POM121 | na |  |  | 2676 | 1.086 | 0.0882 | No |
| 17 | SURF1 | na |  |  | 2718 | 1.075 | 0.0955 | No |
| 18 | GTF3C5 | na |  |  | 2774 | 1.058 | 0.1018 | No |
| 19 | POLA1 | na |  |  | 2786 | 1.055 | 0.1105 | No |
| 20 | REV3L | na |  |  | 3013 | 1.001 | 0.1074 | No |
| 21 | SNAPC4 | na |  |  | 3022 | 0.999 | 0.1158 | No |
| 22 | ADCY6 | na |  |  | 3056 | 0.990 | 0.1227 | No |
| 23 | NT5C | na |  |  | 3213 | 0.956 | 0.1229 | No |
| 24 | ZWINT | na |  |  | 3236 | 0.954 | 0.1301 | No |
| 25 | STX3 | na |  |  | 3386 | 0.925 | 0.1304 | No |
| 26 | SUPT5H | na |  |  | 3552 | 0.893 | 0.1296 | No |
| 27 | ZNF707 | na |  |  | 3723 | 0.860 | 0.1282 | No |
| 28 | POLR1C | na |  |  | 4199 | 0.782 | 0.1101 | No |
| 29 | CCNO | na |  |  | 4615 | 0.718 | 0.0946 | No |
| 30 | NPR2 | na |  |  | 4749 | 0.695 | 0.0938 | No |
| 31 | GUK1 | na |  |  | 4987 | 0.654 | 0.0871 | No |
| 32 | CMPK2 | na |  |  | 5304 | 0.604 | 0.0758 | No |
| 33 | RFC2 | na |  |  | 5525 | 0.573 | 0.0692 | No |
| 34 | POLR2E | na |  |  | 5666 | 0.551 | 0.0667 | No |
| 35 | TYMS | na |  |  | 5792 | 0.533 | 0.0648 | No |
| 36 | POLH | na |  |  | 5980 | 0.505 | 0.0595 | No |
| 37 | RFC5 | na |  |  | 6099 | 0.490 | 0.0576 | No |
| 38 | VPS28 | na |  |  | 6306 | 0.462 | 0.0508 | No |
| 39 | POLR3C | na |  |  | 6807 | 0.396 | 0.0280 | No |
| 40 | PRIM1 | na |  |  | 6842 | 0.391 | 0.0297 | No |
| 41 | ITPA | na |  |  | 6935 | 0.381 | 0.0282 | No |
| 42 | PNP | na |  |  | 6968 | 0.376 | 0.0298 | No |
| 43 | HCLS1 | na |  |  | 6985 | 0.373 | 0.0322 | No |
| 44 | POLE4 | na |  |  | 7207 | 0.342 | 0.0236 | No |
| 45 | NFX1 | na |  |  | 7290 | 0.331 | 0.0222 | No |
| 46 | ADA | na |  |  | 7361 | 0.322 | 0.0214 | No |
| 47 | GMPR2 | na |  |  | 7448 | 0.312 | 0.0196 | No |
| 48 | SRSF6 | na |  |  | 8221 | 0.220 | -0.0190 | No |
| 49 | RAD51 | na |  |  | 8268 | 0.214 | -0.0195 | No |
| 50 | ADRM1 | na |  |  | 8420 | 0.199 | -0.0257 | No |
| 51 | UPF3B | na |  |  | 8469 | 0.192 | -0.0265 | No |
| 52 | RFC3 | na |  |  | 8598 | 0.174 | -0.0317 | No |
| 53 | NME4 | na |  |  | 8640 | 0.165 | -0.0324 | No |
| 54 | FEN1 | na |  |  | 8723 | 0.161 | -0.0353 | No |
| 55 | RAE1 | na |  |  | 8849 | 0.141 | -0.0406 | No |
| 56 | XPC | na |  |  | 8906 | 0.135 | -0.0424 | No |
| 57 | SSRP1 | na |  |  | 9282 | 0.091 | -0.0613 | No |
| 58 | GTF2H3 | na |  |  | 9632 | 0.057 | -0.0791 | No |
| 59 | AK1 | na |  |  | 9668 | 0.052 | -0.0805 | No |
| 60 | VPS37D | na |  |  | 9789 | 0.038 | -0.0864 | No |
| 61 | POLD3 | na |  |  | 9852 | 0.029 | -0.0894 | No |
| 62 | TAF13 | na |  |  | 10691 | -0.064 | -0.1328 | No |
| 63 | ERCC1 | na |  |  | 10709 | -0.065 | -0.1332 | No |
| 64 | UMPS | na |  |  | 10737 | -0.071 | -0.1340 | No |
| 65 | CANT1 | na |  |  | 10869 | -0.089 | -0.1400 | No |
| 66 | POLR2D | na |  |  | 10986 | -0.104 | -0.1452 | No |
| 67 | BCAM | na |  |  | 11029 | -0.111 | -0.1465 | No |
| 68 | POLR2J | na |  |  | 11066 | -0.116 | -0.1473 | No |
| 69 | DGUOK | na |  |  | 11466 | -0.157 | -0.1669 | No |
| 70 | APRT | na |  |  | 11564 | -0.170 | -0.1705 | No |
| 71 | POLR3GL | na |  |  | 11631 | -0.179 | -0.1724 | No |
| 72 | VPS37B | na |  |  | 11723 | -0.190 | -0.1755 | No |
| 73 | ERCC5 | na |  |  | 11786 | -0.201 | -0.1770 | No |
| 74 | BOLA2 | na |  |  | 11886 | -0.214 | -0.1803 | No |
| 75 | NME1 | na |  |  | 12003 | -0.217 | -0.1845 | No |
| 76 | GTF2F1 | na |  |  | 12553 | -0.290 | -0.2107 | No |
| 77 | TAF10 | na |  |  | 12617 | -0.301 | -0.2114 | No |
| 78 | POLR2H | na |  |  | 12745 | -0.321 | -0.2152 | No |
| 79 | CSTF3 | na |  |  | 12760 | -0.325 | -0.2131 | No |
| 80 | SNAPC5 | na |  |  | 12811 | -0.331 | -0.2129 | No |
| 81 | POLR1D | na |  |  | 13025 | -0.345 | -0.2210 | No |
| 82 | NUDT21 | na |  |  | 13214 | -0.373 | -0.2276 | No |
| 83 | GTF2H1 | na |  |  | 13241 | -0.376 | -0.2257 | No |
| 84 | ARL6IP1 | na |  |  | 13554 | -0.421 | -0.2383 | No |
| 85 | RFC4 | na |  |  | 13625 | -0.429 | -0.2383 | No |
| 86 | TAF12 | na |  |  | 13644 | -0.433 | -0.2354 | No |
| 87 | SEC61A1 | na |  |  | 13905 | -0.472 | -0.2449 | No |
| 88 | IMPDH2 | na |  |  | 14016 | -0.491 | -0.2464 | No |
| 89 | POLR2C | na |  |  | 14227 | -0.509 | -0.2529 | No |
| 90 | TAF9 | na |  |  | 14283 | -0.516 | -0.2513 | No |
| 91 | CLP1 | na |  |  | 14295 | -0.520 | -0.2473 | No |
| 92 | GPX4 | na |  |  | 14401 | -0.538 | -0.2481 | No |
| 93 | TSG101 | na |  |  | 14435 | -0.546 | -0.2451 | No |
| 94 | DDB1 | na |  |  | 14506 | -0.558 | -0.2438 | No |
| 95 | NME3 | na |  |  | 14825 | -0.608 | -0.2552 | Yes |
| 96 | ZNRD1 | na |  |  | 14925 | -0.623 | -0.2549 | Yes |
| 97 | MPG | na |  |  | 14958 | -0.628 | -0.2511 | Yes |
| 98 | PCNA | na |  |  | 14963 | -0.629 | -0.2458 | Yes |
| 99 | POLB | na |  |  | 14985 | -0.630 | -0.2414 | Yes |
| 100 | BCAP31 | na |  |  | 15001 | -0.633 | -0.2366 | Yes |
| 101 | RALA | na |  |  | 15016 | -0.636 | -0.2318 | Yes |
| 102 | CDA | na |  |  | 15208 | -0.671 | -0.2359 | Yes |
| 103 | MRPL40 | na |  |  | 15341 | -0.694 | -0.2368 | Yes |
| 104 | SMAD5 | na |  |  | 15614 | -0.745 | -0.2445 | Yes |
| 105 | NUDT9 | na |  |  | 15648 | -0.753 | -0.2396 | Yes |
| 106 | EDF1 | na |  |  | 15758 | -0.775 | -0.2386 | Yes |
| 107 | SF3A3 | na |  |  | 15954 | -0.810 | -0.2417 | Yes |
| 108 | RBX1 | na |  |  | 16070 | -0.838 | -0.2404 | Yes |
| 109 | AAAS | na |  |  | 16210 | -0.873 | -0.2400 | Yes |
| 110 | BRF2 | na |  |  | 16244 | -0.884 | -0.2340 | Yes |
| 111 | RPA2 | na |  |  | 16249 | -0.886 | -0.2265 | Yes |
| 112 | RNMT | na |  |  | 16258 | -0.889 | -0.2191 | Yes |
| 113 | USP11 | na |  |  | 16668 | -0.992 | -0.2319 | Yes |
| 114 | NCBP2 | na |  |  | 17024 | -1.096 | -0.2409 | Yes |
| 115 | POLR2G | na |  |  | 17026 | -1.097 | -0.2313 | Yes |
| 116 | RRM2B | na |  |  | 17036 | -1.100 | -0.2221 | Yes |
| 117 | RPA3 | na |  |  | 17084 | -1.110 | -0.2149 | Yes |
| 118 | PDE4B | na |  |  | 17197 | -1.143 | -0.2107 | Yes |
| 119 | TP53 | na |  |  | 17262 | -1.165 | -0.2039 | Yes |
| 120 | ERCC8 | na |  |  | 17282 | -1.175 | -0.1946 | Yes |
| 121 | GTF2H5 | na |  |  | 17336 | -1.195 | -0.1869 | Yes |
| 122 | DUT | na |  |  | 17427 | -1.226 | -0.1809 | Yes |
| 123 | DCTN4 | na |  |  | 17454 | -1.238 | -0.1714 | Yes |
| 124 | TK2 | na |  |  | 17656 | -1.316 | -0.1704 | Yes |
| 125 | TMED2 | na |  |  | 17755 | -1.362 | -0.1636 | Yes |
| 126 | GTF2A2 | na |  |  | 17802 | -1.387 | -0.1539 | Yes |
| 127 | POLR2I | na |  |  | 17823 | -1.398 | -0.1427 | Yes |
| 128 | CETN2 | na |  |  | 18125 | -1.564 | -0.1448 | Yes |
| 129 | POLD4 | na |  |  | 18420 | -1.773 | -0.1446 | Yes |
| 130 | HPRT1 | na |  |  | 18443 | -1.794 | -0.1301 | Yes |
| 131 | EIF1B | na |  |  | 18458 | -1.806 | -0.1150 | Yes |
| 132 | ERCC4 | na |  |  | 18478 | -1.824 | -0.1000 | Yes |
| 133 | COX17 | na |  |  | 18505 | -1.841 | -0.0852 | Yes |
| 134 | GTF2B | na |  |  | 18664 | -2.003 | -0.0760 | Yes |
| 135 | AK3 | na |  |  | 18732 | -2.092 | -0.0611 | Yes |
| 136 | SDCBP | na |  |  | 18811 | -2.209 | -0.0459 | Yes |
| 137 | SUPT4H1 | na |  |  | 18872 | -2.313 | -0.0288 | Yes |
| 138 | POLR2K | na |  |  | 18999 | -2.578 | -0.0128 | Yes |
| 139 | DAD1 | na |  |  | 19019 | -2.642 | 0.0094 | Yes |
Table: GSEA details [plain text format]

  

Fig 2: HALLMARK\_DNA\_REPAIR      
 Blue-Pink O' Gram in the Space of the Analyzed GeneSet

  

Fig 3: HALLMARK\_DNA\_REPAIR: Random ES distribution      
 Gene set null distribution of ES for **HALLMARK\_DNA\_REPAIR**
